# Key biomechanical features of jump-landing under cognitive dual-task conditions: an XGBoost-SHAP-based explainable machine learning analysis

**DOI:** 10.3389/fbioe.2026.1930588

**Published:** 2026-09-09

**Authors:** Yuhang Zhu, Yanlong Zhang, Qingqing Ma, Minting Fang, Ronghe Cui, Xiao Yu, Si Chen

**Affiliations:** 1 College of Sports and Health Sciences, Mudanjiang Normal University, Mudanjiang, China; 2 College of Physical Education, Shenzhen University, Shenzhen, China

**Keywords:** anterior cruciate ligament (ACL), cognitive dual-task, explainable machine learning, jump-landing, SHAP, XGBoost

## Abstract

**Objective:**

This study aims to quantify jump-landing characteristics and compare the performance of four machine learning (ML) models in classifying single- and dual-task conditions. Shapley Additive Explanations (SHAP) was used to identify key biomechanical features distinguishing the conditions and characterize multivariable biomechanical patterns associated with cognitive-motor interference, providing a quantitative basis for understanding associations between cognitive load and jump-landing biomechanics.

**Methods:**

Overall, 240 physically active young men completed single- and dual-task jump-landing tests. Kinematic and ground reaction force data were synchronously collected using a Qualisys high-speed motion capture system and an AMTI force plate. Hip, knee, ankle, and trunk angles were calculated using OpenSim inverse kinematics, and 13 kinematic and landing performance features were extracted. Four ML models were compared within the development set using participant-grouped nested cross-validation repeated five times. The selected model underwent held-out test-set evaluation; SHAP quantified feature contributions to its predictions.

**Results:**

XGBoost achieved the highest mean outer out-of-fold AUC in the development set (0.992 ± 0.002). Corrected paired comparisons supported higher AUCs for XGBoost than for the three comparator models (all Holm-adjusted P < 0.001). XGBoost’s held-out test-set accuracy and AUC were 0.924 and 0.978, respectively. The five highest-contributing features were the normalized base-of-support ratio (BOS), hip flexion angle at initial contact (HF-IC), trunk flexion range of motion (TF-ROM), ankle dorsiflexion/plantarflexion angle at initial contact (PF-IC), and trunk flexion angle at initial contact (TF-IC). Under the dual-task condition, BOS and TF-ROM increased, whereas HF-IC, PF-IC, and TF-IC decreased, characterizing task-condition differences.

**Conclusion:**

The XGBoost-SHAP model identified and quantified key biomechanical changes during cognitive dual-task jump-landing, providing an explainable, quantitative description of dual-task landing biomechanics. These changes may be related to competition for limited attentional resources between motor control and cognitive tasks. The following five core features collectively characterize the biomechanical pattern distinguishing dual-task from single-task landing: increased BOS, decreased HF-IC, decreased PF-IC, increased TF-ROM, and decreased TF-IC. The model classified task condition only and was developed using data from physically active young men performing a laboratory mental-arithmetic task. Any injury-related interpretation requires prospective validation; generalization to other populations and task contexts requires external validation.

## 1 Introduction

Jump-landing is a typical functional movement task. Because it reflects an individual’s trunk control, lower extremity alignment, and impact absorption strategies under dynamic conditions, it is widely used in sports injury screening and lower limb movement control research (Sayers et al., 2023). Several aberrant movement patterns during landing may be associated with lower extremity injuries. These patterns include altered trunk neuromuscular control, insufficient flexion cushioning, and bilateral asymmetry (Bocheng et al., 2024; Muramoto and Kuruma, 2024). These patterns represent important risk factors for anterior cruciate ligament (ACL) injury (Ericksen et al., 2016).

However, jump-landing in real-world sports scenarios is rarely executed as an isolated movement. Instead, it is frequently accompanied by cognitive activities such as visual search, information processing, decision-making, and attention shifting (Wilke et al., 2021). Athletes often must focus on opponents, equipment, scoring, or other secondary tasks while performing a jump-landing. Therefore, they frequently operate in a cognitive-motor dual-task state (Lin et al., 2020). In this state, limited attentional resources are reallocated. This reallocation can affect postural regulation, movement coordination, and dynamic stability (Zhang et al., 2023). Previous studies show that receiving visual signals during jump-landing, compared to knowing the direction in advance, significantly increases the peak knee valgus angle at the instant of landing. This change is accompanied by a greater knee adduction moment and vertical ground reaction force (GRF) (Saadat et al., 2025). Additionally, as cognitive task difficulty increases, participants exhibit decreased knee flexion angles, insufficient forward trunk lean, reduced bilateral lower limb symmetry, and lower impact absorption efficiency (Dai et al., 2018). This “stiff landing” pattern reduces the energy dissipation time within the muscle-tendon system, forcing ligament structures to bear greater loads. Thus, during dual-task performance, cognitive tasks competitively occupy limited attentional resources. This alters landing mechanics, leading to stiffer landing strategies and poorer stability control. These changes align with the biomechanical characteristics associated with ACL injury (Ellenberger et al., 2021). Consequently, under cognitive dual-task conditions, it is highly important to identify the key movement features that distinguish single-task and dual-task jump-landing conditions and to evaluate them objectively.

Regarding landing assessment, the Landing Error Scoring System (LESS) evaluates jump-landing quality using itemized “movement errors.” LESS is widely used for movement quality assessment, injury risk screening, and intervention monitoring because it is simple, low-cost, and has good reliability and validity. However, existing studies mostly focus on the total LESS score, single items, or a few specific metrics, which limits the scope of analysis. Furthermore, most jump-landing assessments are conducted under single-task conditions. They neglect the cognitive-motor dual-task state common in real sports, making it difficult to reflect true changes in trunk control and landing cushioning strategies during distraction. The expert consensus-based “clinical assessment scale for the vertical drop jump” also provides a standardized framework for ACL-related evaluations (Gagnon et al., 2017). These tools typically combine frontal and sagittal views to score key indicators such as trunk control, lower extremity alignment, knee flexion angle, and landing stability. However, the scoring still relies on the evaluator’s subjective judgment of joint angles and trunk postures from 2D videos. This subjective approach may not adequately capture complex biomechanical interactions in real competition or training (Chijimatsu et al., 2023). This limitation suggests that in a dual-task context, it remains unclear which features best distinguish the two task conditions, as well as the relative contributions of these features. Therefore, in complex scenarios involving multi-joint and multi-planar coordinated changes, traditional methods suffer from limited analysis dimensions and a lack of individual-level interpretability. They have limited capacity to characterize individual-level multi-joint coupling and nonlinear biomechanical patterns induced by dual-tasking. Ultimately, previous research has not systematically identified the key movement features that best differentiate the two task conditions, nor has it determined the relative contributions of each feature.

Machine learning (ML) models can automatically learn patterns from data, reveal complex nonlinear relationships among input features, and predict new data. Therefore, they are widely applied in various fields (He et al., 2023). Currently, commonly used ML models mainly include eXtreme Gradient Boosting (XGBoost), Support Vector Machines (SVM), Logistic Regression, and Random Forest. Combining these models with Shapley Additive Explanations (SHAP) helps explain prediction results. This approach identifies global feature importance and local explanations, thereby capturing complex nonlinear biomechanical features related to injury (Kokkotis et al., 2022). In terms of algorithm selection, ensemble learning methods are widely adopted due to their strong capability to handle high-dimensional data, robustness to missing values, and relatively high interpretability (Musat et al., 2024). Recent research covers multiple areas, including athletic performance analysis, injury prediction, tactical decision support, and training optimization. These advancements are changing how athletes train, compete, and recover, while validating the predictive superiority of ML models through multi-algorithm comparisons (Yuan et al., 2025). For example, a study of collegiate athletes from basketball, football, soccer, and volleyball used lower-extremity strength, postural-stability, flexibility, previous-injury, and demographic measures to develop a Random Forest model of lower-extremity musculoskeletal injury risk (Henriquez et al., 2020). Meanwhile, SVM and deep learning models, such as Long Short-Term Memory (LSTM) networks, have also shown great potential. Due to their advantages in processing time-series data and high-dimensional features, they are used to analyze and predict overuse injuries (Nuyts et al., 2022; Zhang and Chen, 2024). Additionally, ML techniques are applied to predict match outcomes and identify variations in movement patterns. In tennis, ML can accurately predict player scores (Zhao et al., 2024). For rugby, XGBoost achieves the highest accuracy in predicting match outcomes (Sreeja et al., 2025). Ultimately, ML can extract insights from complex sports data to evaluate athletes’ skills, abilities, and individual performance tendencies (Davis et al., 2024). Taken together, XGBoost has particular strengths in complex multivariable classification. Its regularized gradient-boosting framework can capture nonlinear relationships while controlling model complexity (Chen and Guestrin, 2016). These characteristics provided the methodological rationale for including XGBoost as one of the candidate algorithms evaluated in this study.

For athletic performance analysis, injury prediction, and tactical decision support, ML models can integrate multiple risk factors. They have demonstrated great potential, and effective predictive models have been developed (Leckey et al., 2025). Beyond injury prediction, ML has also been applied directly to laboratory biomechanical data. For vertical jumping, an artificial neural network estimated lower-extremity joint torques from ground-reaction-force measurements and related parameters. The model was evaluated against inverse-dynamics results (Liu et al., 2009). In gait analysis, ML surrogate models used optical motion-capture and musculoskeletal-modeling variables to predict musculoskeletal-model-derived medial and lateral knee contact forces in near real time (Giarmatzis et al., 2020). SHAP-based explainable ML has also been applied to the biomechanical classification of ACL-related gait patterns (Kokkotis et al., 2022). However, these studies focused on biomechanical estimation or classification rather than the multivariable characterization of cognitive dual-task jump-landing. It remains unclear whether multiple biomechanical features jointly distinguish single-task from dual-task landing and how individual features contribute to this multivariable pattern. Therefore, the purpose of this study is to apply established ML and SHAP methods to identify and interpret the multivariable biomechanical patterns that distinguish the two task conditions. Biomechanical features will be quantified through kinematic data collection, and ML models using different algorithms will be developed and compared. The best-performing model will be selected and combined with explainable methods, such as SHAP, to quantitatively analyze the contribution of each feature in distinguishing the two task conditions. Based on this, we hypothesized that XGBoost would outperform the other models in classifying the two task conditions because it can accommodate nonlinear multivariable relationships among complex biomechanical factors. Furthermore, we hypothesized that explainable ML models could identify and quantify key biomechanical features under dual-task conditions, manifested as changes in the normalized base-of-support ratio and the postures of the trunk, hip, and ankle joints at initial contact (IC).

## 2 Methods

### 2.1 Participants

**FIGURE 1:**
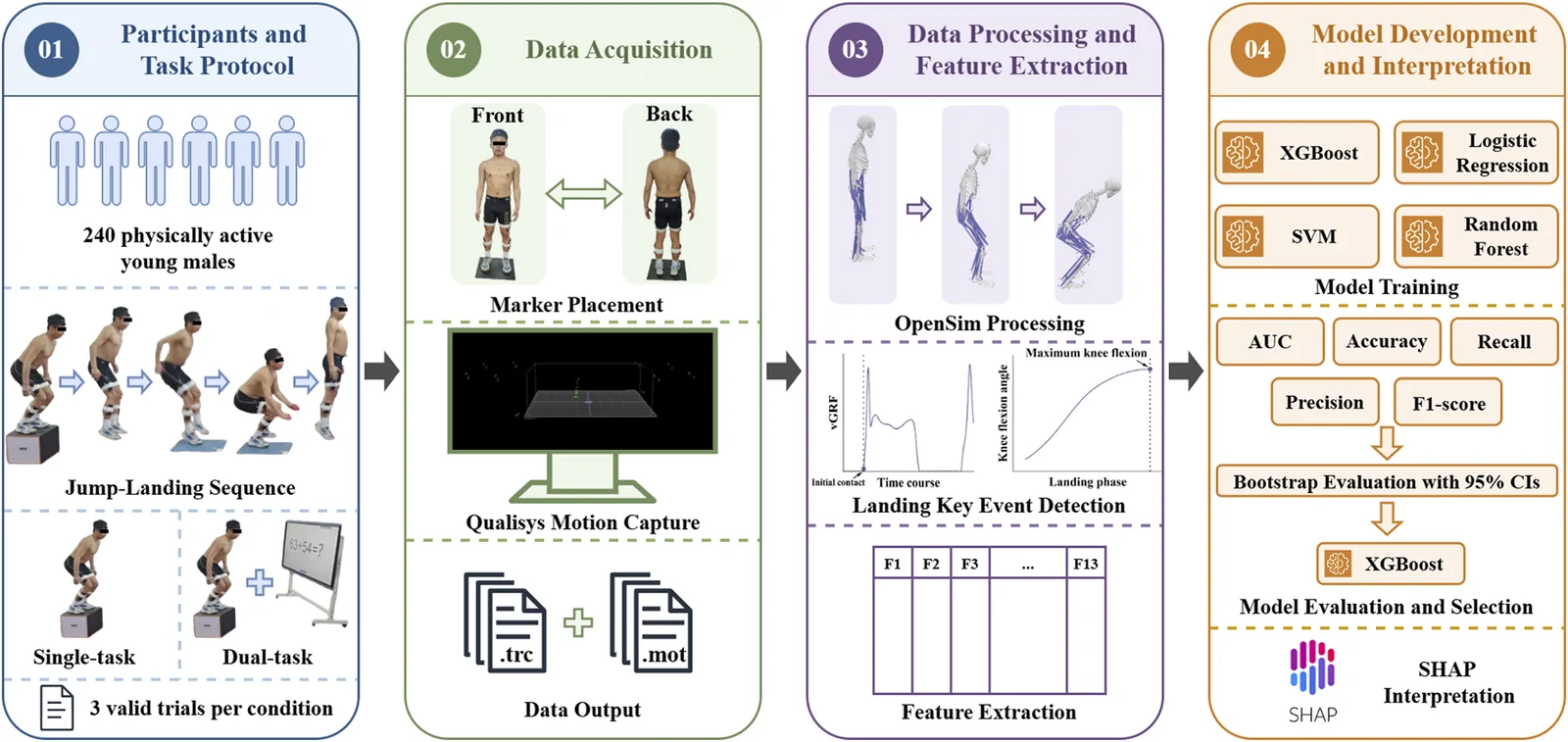
Overview of the research workflow.

A total of 240 physically active young men voluntarily participated in this study (age: 22.0 ± 2.5 years; height: 173.4 ± 3.7 cm; weight: 83.8 ± 7.3 kg). All participants had engaged in regular physical activity for at least the previous 3 years, with a minimum frequency of three sessions per week. Participants had no history of major trunk or lower extremity injuries, neurological disorders, or musculoskeletal conditions that could affect jump-landing performance. This study was approved by the Ethics Committee of Mudanjiang Normal University (Approval No. 2025008) and was conducted in accordance with the Declaration of Helsinki. Prior to participation, all participants were fully informed of the study procedures, testing protocol, and potential risks and provided written informed consent. The overall study workflow is illustrated in Figure 1.

### 2.2 Data collection

**FIGURE 2:**
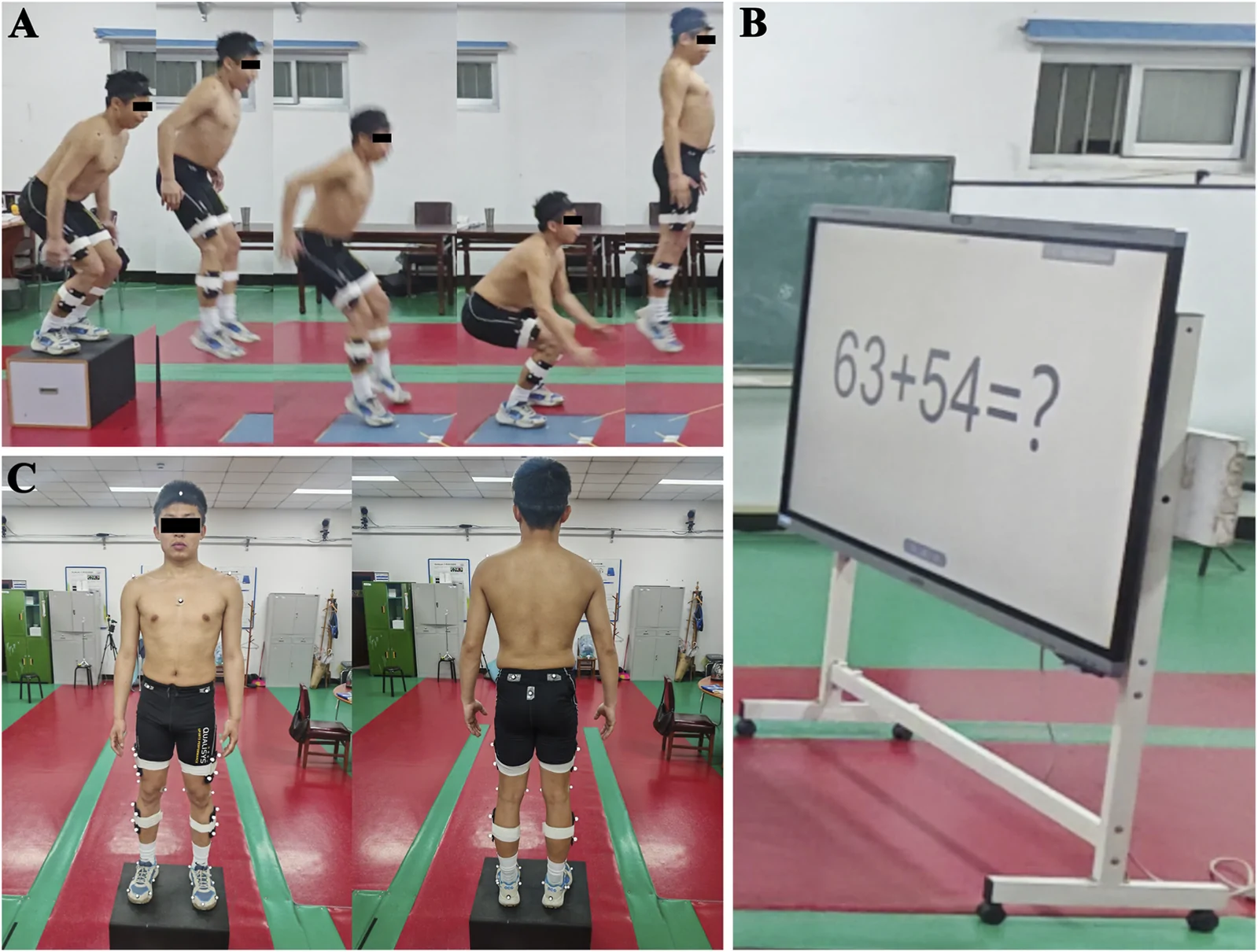
Experimental setup and data collection. **(A)** Jump-landing task sequence illustrating the drop vertical jump protocol. **(B)** Cognitive dual-task stimulus presented via screen prior to each dual-task trial (two-digit mental arithmetic). **(C)** Anterior and posterior views of reflective marker placement.

In this study, an eight-camera high-speed motion capture system (Qualisys, 200 Hz) and a force plate (AMTI 400600, 1000 Hz) were used to synchronously collect single-task and dual-task jump-landing data. The systems were synchronized in Qualisys Track Manager, and their data were recorded on a common time base. Reflective markers were placed according to anatomical landmarks on the lower extremity, pelvis, trunk, and feet. Figure 2 illustrates the jump-landing workflow, the presentation of the dual-task cognitive stimulus, and the marker placement. The cameras and force plate recorded the three-dimensional (3D) marker trajectories and GRF data during the jump-landing, respectively. The former were used to reconstruct the kinematic changes of the hip, knee, ankle, and trunk, while the latter were used to identify the timing of IC.

Prior to formal testing, all participants performed a standardized warm-up and familiarized themselves with the single-task and dual-task jump-landing protocols under the guidance of researchers. Participants first completed several practice trials to ensure a full understanding of the movement and cognitive task requirements. The dominant leg was recorded before testing and was defined as the leg preferred for kicking a ball. For the jump-landing task, participants stood on a 30-cm-high box and jumped forward, landing with their feet at a distance of approximately 50% of their body height from the front edge of the box. Specifically, the dominant leg was required to land in the center area of the force plate, followed immediately by a maximal effort vertical jump upon landing.

Under the dual-task condition, participants executed a two-digit mental addition task while performing the same jump-landing movement. This task imposed a concurrent cognitive demand during the jump-landing movement. Before each dual-task trial, a two-digit addition problem was presented on a screen, with both addends ranging from 50 to 99. Participants were required to perform the mental arithmetic as accurately as possible during the jump-landing and report their answer to the staff immediately after the trial.

During testing, response time was not recorded. Participants were instructed to give equal attention to the motor and cognitive tasks and to perform both tasks to the best of their ability. An arithmetic response was recorded as correct if the reported sum matched the correct answer and as incorrect otherwise. Incorrect responses did not invalidate the trial. Throughout the testing process, a trial was considered invalid and repeated if any of the following situations occurred: an obvious loss of balance, failure to complete the movement as required, the dominant foot failing to land entirely within the effective collection area of the force plate, hands touching the ground after landing, failure to provide any arithmetic answer after a dual-task trial, or severe loss of key marker trajectories at IC. Only the overall number of invalid and repeated trials was recorded; trial-level information on task condition, specific reason(s) for invalidation, and whether multiple criteria were met was not recorded. To ensure data stability and reliability, three valid trials were collected for each participant under both single-task and dual-task conditions. The condition order was randomized to minimize order effects. To minimize fatigue, participants rested between trials until they felt ready to continue, typically for about 1 min.

Following the trials, marker trajectories were identified, named, checked, and gap-filled in Qualisys Track Manager. The 3D marker trajectories and force plate data were then exported for subsequent OpenSim inverse kinematics analysis and feature extraction. The gap-filled 3D marker trajectories and GRF data were filtered using a fourth-order zero-phase Butterworth low-pass filter. The cutoff frequencies were set at 12 Hz for marker trajectories and 50 Hz for GRF data (Blackburn and Padua, 2009; Imai et al., 2022).

### 2.3 Data processing and feature extraction

After data collection, the processed 3D marker trajectories and force-plate data from Qualisys Track Manager were converted into OpenSim-compatible marker-trajectory (.trc) and ground-reaction-force (.mot) files, respectively. During conversion, both datasets were expressed in the standard OpenSim coordinate system. The positive X-axis was aligned with the direction of progression. The positive Y-axis was directed vertically upward, and the positive Z-axis was directed to the participant’s right. The ground-reaction-force files were used to identify the instant of IC. OpenSim 4.4 was used for model scaling and inverse-kinematics analysis. The Gait2392 musculoskeletal model was used. It has 23 degrees of freedom in the trunk and lower extremities and includes 92 muscle actuators (Yap et al., 2021). A static calibration trial was used to scale the model to each participant with the OpenSim Scale Tool. Segment dimensions were adjusted based on the distances between corresponding experimental and model markers. Total model mass was matched to the participant’s measured body mass. Joint-angle time series for the hip, knee, ankle, and trunk were then obtained from the inverse-kinematics analysis. Scaling and inverse-kinematics quality were assessed using the RMS and maximum marker errors reported by OpenSim. These marker-error measures served as the principal quantitative verification criteria for model scaling and inverse kinematics in the present workflow; no additional independent model-verification analysis was performed. For model scaling, both the RMS and maximum marker errors were required to be below 1 cm. For inverse kinematics, the RMS and maximum marker errors were required to be below 2 and 4 cm, respectively. All scaled models and retained trials met these criteria.

The calculated angles were defined according to the coordinate conventions of the Gait2392 model. Hip angles represented femoral motion relative to the pelvis. Knee angles represented tibial motion relative to the femur. Ankle angles represented talar motion relative to the tibia. Trunk angles represented torso motion relative to the pelvis. To ensure consistent interpretation of joint angle directions, the numerical signs for movement directions were uniformly defined. In the sagittal plane, hip, knee, and trunk flexion were defined as positive, whereas extension was defined as negative. For the ankle, dorsiflexion was defined as positive and plantarflexion as negative. In the frontal plane, adduction or inversion was defined as positive, while abduction or eversion was negative. In the transverse (horizontal) plane, internal rotation was defined as positive, and external rotation was negative.

The instant of IC was defined as the moment when the filtered vertical GRF of the dominant leg first exceeded 10 N (Zamankhanpour et al., 2023). The moment of maximal knee flexion was defined as the moment when the knee flexion angle reached its peak during the post-IC cushioning phase. Based on these two time points, 13 kinematic and landing performance variables were extracted as input features for subsequent ML models. These variables included 10 continuous variables and 3 binary variables. Continuous variables primarily reflected lower limb and trunk postures during the IC phase, as well as joint angle changes during the cushioning phase. Binary variables described the presence or absence of specific landing characteristics. Joint and trunk angle variables were obtained from the OpenSim inverse kinematics output. Spatial or performance variables, such as the normalized base-of-support ratio (BOS) and foot contact symmetry, were calculated or determined based on the.trc marker coordinates. At the GRF-defined IC, BOS was calculated as the ratio of the mediolateral distance between the bilateral medial midfoot markers to the distance between the bilateral shoulder markers, yielding a dimensionless value. The midpoint between the medial and lateral femoral epicondyle markers was defined as the knee midpoint. KMD-IC was coded as present when this midpoint was medial to the ipsilateral medial-midfoot marker at IC. Using the GRF-defined IC as a temporal reference, the contact event for each heel and great-toe marker was defined as the first frame after descent in which its vertical velocity changed from negative to non-negative. For each foot, the earlier marker event defined first foot contact, and the heel–toe event order defined the initial contact pattern. FCS was coded as present when the bilateral first-contact times differed by at least one motion-capture frame (5 ms) or when the initial contact patterns differed; otherwise, it was coded as absent (Padua et al., 2009; Lim et al., 2020). KV-ROM was coded as present when the knee midpoint reached or moved medial to the great-toe marker between IC and maximum knee flexion. The model input features and their definitions are shown in Table 1.

**TABLE 1:** Input features used in the machine learning models.

Feature	Description
KF-IC	Knee flexion angle at initial contact
HF-IC	Hip flexion angle at initial contact
TF-IC	Trunk flexion angle at initial contact
TLF-IC	Trunk lateral flexion angle at initial contact
PF-IC	Ankle dorsiflexion(+)/plantarflexion(−) angle at initial contact
BOS	Ratio of foot stance width to shoulder width at initial contact
FP-IC	Foot rotation angle at initial contact
KF-ROM	Change in knee flexion angle from initial contact to maximum knee flexion
HF-ROM	Change in hip flexion angle from initial contact to maximum knee flexion
TF-ROM	Change in trunk flexion angle from initial contact to maximum knee flexion
KMD-IC	Presence of medial knee valgus position at initial contact, determined by whether the knee midpoint was positioned medial to the midfoot; 0 = absent, 1 = present
FCS	Presence of asymmetric initial foot contact, determined by whether the two feet contacted the ground asynchronously or with different initial contact patterns; 0 = absent, 1 = present
KV-ROM	Presence of knee valgus displacement from initial contact to maximum knee flexion, determined by whether the knee midpoint moved medially and passed through or medial to the great toe; 0 = absent, 1 = present

Trial-level reliability was assessed separately within each task condition. Continuous features were evaluated using ICC(A,3) from a two-way mixed-effects, absolute-agreement, average-measures model, with 95% CIs calculated from the F distribution (Koo and Li, 2016). Binary-feature agreement was evaluated using Gwet’s AC1 with analytic 95% CIs and mean pairwise percentage agreement, defined as the average agreement across the three possible trial pairs (Gwet, 2008). The three valid trials were then aggregated to reduce dependence on any single trial and obtain a representative participant-level value for each condition. For continuous variables, the average value of the three trials was calculated. For binary variables, a majority rule was applied: if a binary feature occurred two or more times across the three trials, it was recorded as 1; otherwise, it was recorded as 0. This approach limits dependence on a single trial and is consistent with established three-trial LESS calculation methods (Padua et al., 2009; Hanzlíková and Hébert-Losier, 2020). Ultimately, each participant generated one representative sample under the single-task condition and one under the dual-task condition for subsequent statistical analysis and ML modeling.

### 2.4 Machine learning model development and evaluation

This study compared four supervised ML classification models: XGBoost, Random Forest, SVM, and Logistic Regression. Task type was used as the ML classification label, where single-task samples were coded as 0 and dual-task samples were coded as 1. Each participant generated one representative sample under the single-task condition and one under the dual-task condition. Thus, the final dataset used for model construction contained 480 movement samples (240 single-task and 240 dual-task samples). To prevent participant-level data leakage, the dataset was divided once at the participant level into a development set and a held-out test set at a 7:3 ratio using a random seed of 42. Both task-condition samples from each participant were assigned to the same data partition. The development set contained 168 participants and 336 samples, whereas the held-out test set contained 72 participants and 144 samples. Model comparison, hyperparameter optimization, and algorithm selection were performed exclusively within the development set, whereas the held-out test set was reserved for final evaluation of the selected algorithm.

Within the development set, the four models were evaluated using nested cross-validation implemented with five-fold StratifiedGroupKFold in both the outer and inner loops and repeated five times. Each repetition comprised five outer folds for performance estimation and five inner folds for grid-search hyperparameter optimization. Mean area under the receiver operating characteristic curve (AUC) across the inner folds was used for hyperparameter optimization, whereas the mean AUC calculated from the complete outer out-of-fold predictions across the five repetitions was used for algorithm selection. Subject ID was used as the grouping variable in both loops, ensuring that both samples from each participant remained within the same fold. Within each repetition, the same outer-fold assignments were used for all four models. The hyperparameter configuration selected in the inner loop was subsequently evaluated on the corresponding outer validation fold. For Logistic Regression and SVM, continuous predictors were standardized using a StandardScaler fitted exclusively within each training fold as part of a scikit-learn Pipeline, whereas binary predictors retained their original 0/1 coding. Feature scaling was not applied to Random Forest or XGBoost. No missing predictor values were present; therefore, no missing-value imputation was performed. No supervised feature selection was performed, and all 13 input features were retained. The hyperparameter search spaces and final selected values are presented in Table 2.

**TABLE 2:** Hyperparameter search spaces and selected values for the machine-learning algorithms.

Model	Hyperparameter	Search space	Selected value
Logistic regression	C	0.01, 0.1, 1, 10, 100	10
​	Penalty	L1, L2	L2
SVM	C	0.1, 1, 10, 100	10
​	Gamma	0.001, 0.01, 0.1	0.01
​	Kernel	Linear, poly, rbf, sigmoid	rbf
Random forest	n_estimators	100, 200, 400, 500	400
​	max_depth	2, 3, 4, 6	4
​	min_samples_split	2, 5, 10	2
​	min_samples_leaf	1, 2, 4, 6	2
XGBoost	n_estimators	100, 200, 400, 500	400
​	max_depth	2, 3, 4, 6	6
​	learning_rate	0.01, 0.03, 0.05, 0.1	0.03
​	reg_alpha	0.01, 0.1, 0.3, 0.5	0.3
​	reg_lambda	0.5, 1.0, 1.5, 2.0	1.0

Model performance was evaluated using accuracy, recall, precision, F1-score, and AUC. Accuracy represents the proportion of correctly classified samples overall. Recall indicates the model’s ability to identify dual-task samples. Precision denotes the proportion of true dual-task samples among those predicted as dual-task. The F1-score comprehensively reflects the balance between precision and recall. The AUC was used to quantify the overall discriminative ability of the model to distinguish between single-task and dual-task samples. The specific formulas are as follows:

$$Accuracy=\frac{TP+TN}{TP+TN+FP+FN}$$ (1)

$$Precision=\frac{TP}{TP+FP}$$ (2)

$$Recall=\frac{TP}{TP+FN}$$ (3)

$$F1-score=2\times \frac{Precision\times Recall}{Precision+Recall}$$ (4)

where TP, TN, FP, and FN represent true positives, true negatives, false positives, and false negatives, respectively.

Classification thresholds were 0.50 for Logistic Regression, Random Forest, and XGBoost and 0 for the SVM decision function. A base random seed of 42 was used, with deterministic seeds varied by repetition, outer fold, and model. For each repetition, predictions from the five outer validation folds were combined to obtain complete outer out-of-fold predictions for the development set. AUC, accuracy, recall, precision, and F1-score were calculated separately from the complete predictions obtained in each repetition. The resulting five values for each metric were summarized as the mean and standard deviation. Because identical outer-fold assignments were used for all four models, paired AUC differences were calculated within each outer validation fold as the AUC of XGBoost minus that of the comparator. This yielded 25 matched differences for each comparison. Mean outer-fold paired AUC differences, corrected 95% confidence intervals, t statistics, and two-sided P values were calculated using the corrected repeated k-fold t-test (Nadeau and Bengio, 2003). Holm adjustment was applied across the three prespecified comparisons.

The algorithm with the highest mean complete outer out-of-fold AUC across the five repetitions was selected. Its final hyperparameters were determined on the complete development set using participant-grouped five-fold cross-validation. The final model was then fitted using all development-set samples. The fixed final model was evaluated on the held-out test set. Point estimates of test-set AUC, accuracy, recall, precision, and F1-score were calculated from the original test set. Percentile-based 95% confidence intervals were obtained from 1,000 participant-level bootstrap resamples. Both task-condition samples from each sampled participant were retained together, and the model was not refitted during bootstrap resampling. These intervals reflect uncertainty arising from test-set composition for the fixed final model. They do not capture variability arising from model training, hyperparameter optimization, or participant allocation. The ML analyses were performed in Python 3.13 using NumPy 2.4.2, pandas 3.0.0, scikit-learn 1.7.0, SciPy 1.17.0, and XGBoost 3.1.3.

### 2.5 Model interpretation

To interpret the ML model predictions, SHAP was applied for model explanation. SHAP is a feature contribution analysis method based on cooperative game theory. It quantifies the contribution of each input feature to the model prediction and reflects its positive or negative impact on the prediction direction. By ranking these feature contributions, SHAP identifies the key kinematic and landing performance features the model relies on to distinguish between single-task and dual-task jump-landing conditions. The formula for calculating the SHAP value is as follows:

$$\phi_{i}=\sum_{S\subseteq N\setminus \left\{i\right\}}\frac{\left|S\right|!\left(\left|N\right|-\left|S\right|-1\right)!}{\left|N\right|!}\left[f\left(S\cup \left\{i\right\}\right)-f\left(S\right)\right]$$ (5)

In this equation, $\phi_{i}$ represents the SHAP value of the i -th feature, S is a subset of features, N is the set of all input features, and $f\left(S\right)$ represents the model output based on the feature subset S . Model comparison and selection were completed within the development set. SHAP was then applied to explain predictions from the selected XGBoost model on the held-out test set. SHAP values were calculated using TreeExplainer in SHAP 0.50, with model_output set to “raw”. Under the default feature_perturbation = “auto” setting, TreeExplainer used the tree-path-dependent approach because no background dataset was explicitly supplied. For this binary classifier, the raw output represents the log-odds of the positive class (dual-task, coded as 1). The corresponding predicted probabilities were obtained using the inverse-logit transformation. After calculating the SHAP values, the model output is decomposed into the additive contributions of individual features. This approach clarifies both the magnitude and direction of each feature’s contribution to the model output. Consequently, it provides a quantitative basis for describing the features used by the model to distinguish the two task conditions.

### 2.6 Statistical analysis

Statistical analysis was performed using SPSS 21.0. Effect estimates, 95% CIs, and Holm-adjusted P values were calculated using Python 3.13. Continuous variables were expressed as mean ± standard deviation or median (25th percentile, 75th percentile), as appropriate. Within-participant paired differences were calculated as dual-task minus single-task values. Their normality was assessed using the Shapiro–Wilk test. Normally distributed paired differences were compared using paired-samples *t* tests. Non-normally distributed paired differences were compared using Wilcoxon signed-rank tests. Binary variables were expressed as frequencies (percentages), and the McNemar test was used to compare the differences in feature proportions between the two task conditions. Cohen’s $d_{z}$ was reported for paired-samples *t* tests, with 95% CIs calculated using the noncentral- t method. Matched-pairs rank-biserial correlation was reported for Wilcoxon signed-rank tests, with 95% CIs calculated using a Fisher-transformation normal approximation. Paired differences in proportions were reported for McNemar tests, with 95% CIs calculated using the Newcombe score method for paired data. P values for the 13 feature comparisons were adjusted using the Holm procedure. All effect estimates were oriented as dual-task minus single-task. All statistical tests were two-tailed, and Holm-adjusted P < 0.05 was considered statistically significant.

## 3 Results

### 3.1 Descriptive statistics of feature variables

Across both task conditions, 273 trials met one or more of the predefined invalid-trial criteria described above and were repeated. Among the 720 valid dual-task trials, 461 arithmetic responses were correct and 259 were incorrect (accuracy, 64.0%; error rate, 36.0%). After aggregation of the three valid trials within each condition, the final dataset comprised 480 representative samples from 240 participants, with 240 samples per condition. Feature-specific reliability and agreement results are presented in Tables 3, 4. ICC(A,3) values ranged from 0.763 to 0.946 in the single-task condition and from 0.753 to 0.932 in the dual-task condition. Gwet’s AC1 values ranged from 0.833 to 0.991 and from 0.810 to 0.971, respectively. Mean pairwise agreement ranged from 91.4% to 99.2% in the single-task condition and from 86.1% to 97.5% in the dual-task condition. Table 5 presents the descriptive statistics and paired comparison results for all 13 feature variables. After Holm adjustment, all continuous variables except TLF-IC and FP-IC showed significant differences between the two conditions. The three binary variables showed no significant differences between the two conditions.

**TABLE 3:** Within-session reliability of continuous biomechanical features across three valid trials.

Variable	Single-task ICC (A,3) (95% CI)	Dual-task ICC (A,3) (95% CI)
KF-IC	0.922 (0.903, 0.938)	0.927 (0.909, 0.942)
HF-IC	0.928 (0.911, 0.942)	0.895 (0.870, 0.916)
TF-IC	0.888 (0.861, 0.910)	0.928 (0.911, 0.942)
TLF-IC	0.763 (0.706, 0.811)	0.753 (0.693, 0.803)
PF-IC	0.930 (0.913, 0.944)	0.928 (0.911, 0.942)
BOS	0.928 (0.911, 0.942)	0.875 (0.845, 0.900)
FP-IC	0.903 (0.880, 0.922)	0.926 (0.908, 0.941)
KF-ROM	0.847 (0.810, 0.878)	0.820 (0.777, 0.856)
HF-ROM	0.912 (0.891, 0.930)	0.932 (0.916, 0.946)
TF-ROM	0.946 (0.933, 0.957)	0.911 (0.890, 0.929)

ICC (A,3) denotes a two-way mixed-effects, absolute-agreement, average-measures model and reflects the reliability of the mean of the three valid trials. CI, confidence interval.

**TABLE 4:** Within-session agreement of binary biomechanical features across three valid trials.

Variable	Single-task		Dual-task	
	AC1 (95% CI)	Agreement (%)	AC1 (95% CI)	Agreement (%)
KMD-IC	0.991 (0.981, 1.000)	99.2	0.971 (0.952, 0.990)	97.5
FCS	0.833 (0.777, 0.890)	91.7	0.810 (0.750, 0.870)	90.3
KV-ROM	0.896 (0.858, 0.934)	91.4	0.816 (0.762, 0.869)	86.1

**TABLE 5:** Descriptive statistics and paired comparisons of kinematic and landing performance features under single-task and dual-task conditions.

Variable	Single-task (0)	Dual-task (1)	Effect measure (95% CI)	Holm-adjusted *P*
KF-IC (°)	33.62 ± 8.13	31.89 ± 7.23	*d*_*z*_ = −0.274 (−0.402, −0.145)	<0.001
HF-IC (°)	21.70 (15.81, 26.84)	14.66 (8.35, 22.37)	*r*_*rb*_ = −0.795 (−0.837, −0.743)	<0.001
TF-IC (°)	19.76 (14.79, 25.81)	19.16 (10.85, 22.70)	*r*_*rb*_ = −0.567 (−0.647, −0.475)	<0.001
TLF-IC (°)	4.25 (2.13, 5.84)	3.73 (2.33, 5.81)	*r*_*rb*_ = 0.030 (−0.097, 0.156)	0.699
PF-IC (°)	6.09 (−3.76, 24.47)	−2.15 (−7.54, 7.76)	*r*_*rb*_ = −0.697 (−0.757, −0.625)	<0.001
BOS	0.99 (0.87, 1.09)	1.20 (1.04, 1.33)	*r*_*rb*_ = 0.984 (0.979, 0.987)	<0.001
FP-IC (°)	−12.94 ± 6.29	−13.31 ± 6.63	*d*_*z*_ = −0.061 (−0.187, 0.066)	0.699
KF-ROM (°)	84.49 (76.96, 90.29)	88.02 (82.04, 93.88)	*r*_*rb*_ = 0.457 (0.351, 0.552)	<0.001
HF-ROM (°)	56.83 ± 14.92	59.57 ± 18.36	*d*_*z*_ = 0.198 (0.070, 0.326)	0.014
TF-ROM (°)	−0.75 (−6.35, 4.95)	4.89 (0.30, 10.84)	*r*_*rb*_ = 0.696 (0.624, 0.756)	<0.001
KMD-IC (%)	​	​	Δ*p* = 3.8 pp (−0.2, 8.0)	0.466
Present (1)	7 (2.9%)	16 (6.7%)	​	​
Absent (0)	233 (97.1%)	224 (93.3%)	​	​
FCS (%)	​	​	Δ*p* = −5.4 pp (−12.8, 2.0)	0.563
Present (1)	120 (50.0%)	107 (44.6%)	​	​
Absent (0)	120 (50.0%)	133 (55.4%)	​	​
KV-ROM (%)	​	​	Δ*p* = 5.0 pp (−0.6, 10.6)	0.466
Present (1)	18 (7.5%)	30 (12.5%)	​	​
Absent (0)	222 (92.5%)	210 (87.5%)	​	​

Data are presented as mean ± SD, median (Q1, Q3), or n (%), as appropriate. Effect measures were calculated as dual-task minus single-task; Δ*p* is expressed in percentage points (pp). *P* values were adjusted across the 13 comparisons using the Holm procedure.

### 3.2 Performance comparison of machine learning models

The four candidate algorithms were compared within the development set using five repetitions of participant-grouped five-fold nested cross-validation (Table 6). XGBoost achieved the highest mean outer out-of-fold AUC (0.992 ± 0.002), followed by Random Forest (0.962 ± 0.002), SVM (0.939 ± 0.003), and Logistic Regression (0.890 ± 0.002). Paired comparisons based on the 25 matched outer-fold AUC differences are presented in Table 7. The mean paired ΔAUCs for XGBoost versus Random Forest, SVM, and Logistic Regression were 0.029 (95% CI: 0.015–0.043), 0.052 (95% CI: 0.027–0.076), and 0.101 (95% CI: 0.071–0.131), respectively. All Holm-adjusted P values were < 0.001. After the final hyperparameters were determined on the complete development set, the final XGBoost model was fitted using all development-set observations and evaluated on the held-out test set. It achieved an AUC of 0.978 (95% CI: 0.952–0.994), accuracy of 0.924 (95% CI: 0.875–0.965), recall of 0.889 (95% CI: 0.808–0.953), precision of 0.955 (95% CI: 0.897–1.000), and F1-score of 0.922 (95% CI: 0.868–0.961).

**TABLE 6:** Development-set performance of the machine-learning algorithms across five repetitions of nested cross-validation (mean ± SD).

Algorithm	AUC	Accuracy	Recall	Precision	F1-score
XGBoost	0.992 ± 0.002	0.951 ± 0.010	0.950 ± 0.015	0.951 ± 0.012	0.951 ± 0.010
Random forest	0.962 ± 0.002	0.886 ± 0.006	0.862 ± 0.015	0.906 ± 0.005	0.883 ± 0.007
SVM	0.939 ± 0.003	0.853 ± 0.011	0.899 ± 0.017	0.823 ± 0.008	0.859 ± 0.011
Logistic regression	0.890 ± 0.002	0.799 ± 0.007	0.760 ± 0.009	0.824 ± 0.006	0.791 ± 0.007

**TABLE 7:** Paired AUC comparisons between XGBoost and comparator algorithms in the development set.

Comparison	ΔAUC (95% CI)	*t* (df)	Holm-adjusted *P*
XGBoost − random forest	0.029 (0.015–0.043)	4.345 (24)	<0.001
XGBoost − SVM	0.052 (0.027–0.076)	4.418 (24)	<0.001
XGBoost − logistic regression	0.101 (0.071–0.131)	6.883 (24)	<0.001

### 3.3 Model explanation

**FIGURE 3:**
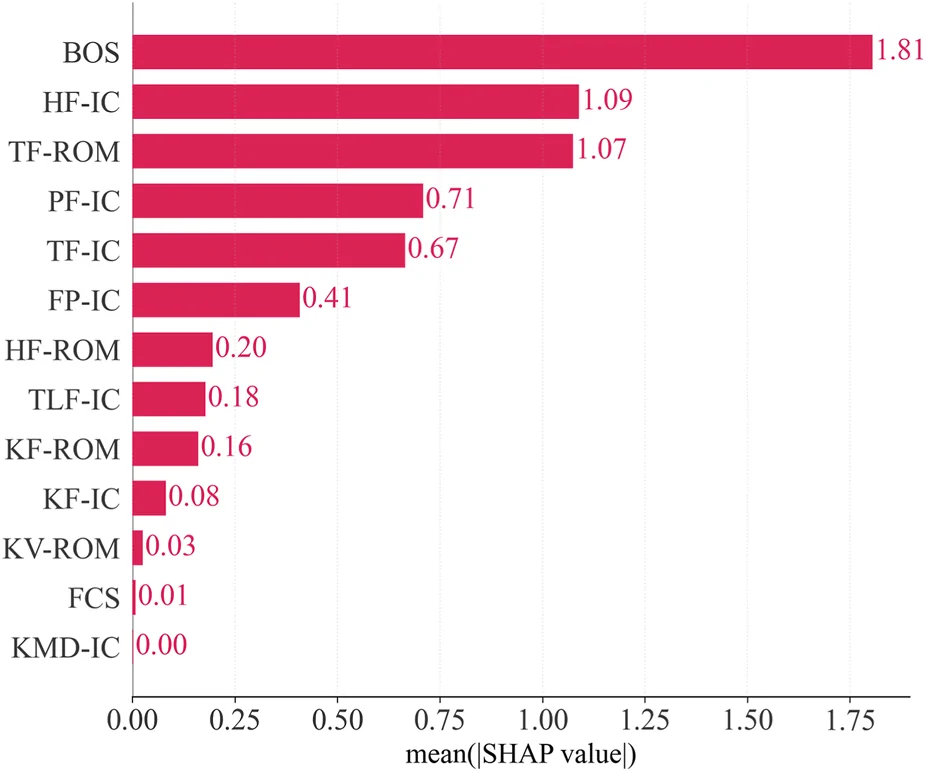
Feature importance ranking derived from SHAP analysis.

SHAP quantified the contribution of each biomechanical feature to the model output. It identified the key features the model relied on to distinguish between the two task conditions. Figure 3 illustrates the feature importance ranking based on mean absolute SHAP values, reflecting the average impact intensity of each feature on model predictions. Five features were identified as the most influential factors: normalized base-of-support ratio (BOS), hip flexion angle at IC (HF-IC), trunk flexion range of motion (TF-ROM), ankle dorsiflexion/plantarflexion angle at IC (PF-IC), and trunk flexion angle at IC (TF-IC). Their mean absolute SHAP values were 1.81, 1.09, 1.07, 0.71, and 0.67, respectively. Meanwhile, all five variables showed statistically significant differences between the two conditions. The SHAP values of the remaining eight features were relatively small. Among them, FP-IC, HF-ROM, KF-ROM, and TLF-IC showed low contributions, while the contributions of KF-IC, KV-ROM, FCS, and KMD-IC were near zero.

**FIGURE 4:**
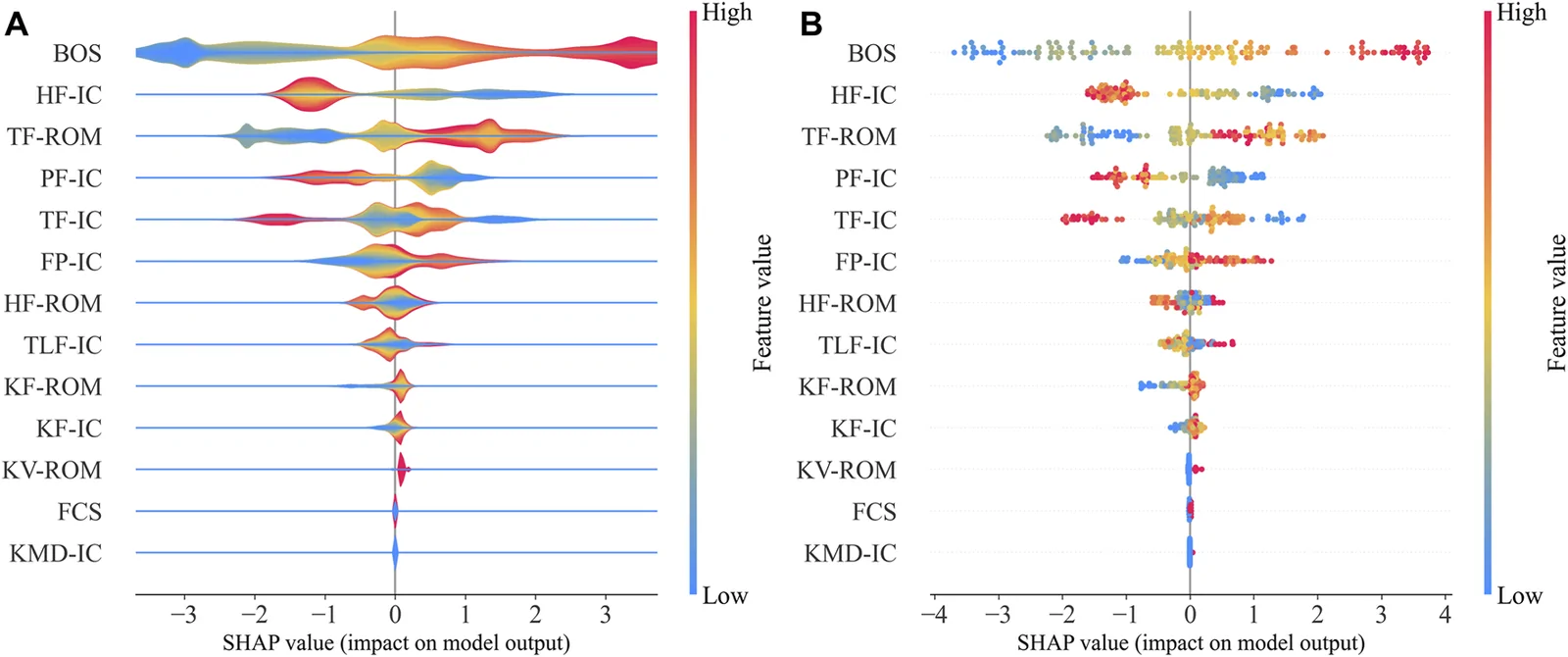
SHAP comprehensive analysis chart. **(A)** Violin plots showing the distribution of SHAP values stratified by feature values. **(B)** Beeswarm plot illustrating SHAP value distributions across features, with point colors indicating original feature magnitudes (red for high, blue for low).

After establishing the feature importance ranking, we combined SHAP-based beeswarm and violin plots to understand the relationships between feature values and their SHAP contributions. These plots demonstrate the direction and magnitude of SHAP contributions across different feature-value ranges. Figure 4 reveals the SHAP value distribution patterns for all features, using color-coding to show the relationship between feature values and SHAP values. The violin plot (Figure 4A) shows inter-individual variability. Specifically, BOS, HF-IC, TF-ROM, PF-IC, and TF-IC exhibit wide SHAP value distributions, reflecting clear individual differences in their contributions to the model output. The beeswarm plot (Figure 4B) shows directional associations and displays the distribution of individual samples, where dot colors represent feature levels. A clear, regular pattern emerges between feature levels and their corresponding SHAP contribution directions, reflecting the different contributions of features to the direction of the model output.

**FIGURE 5:**
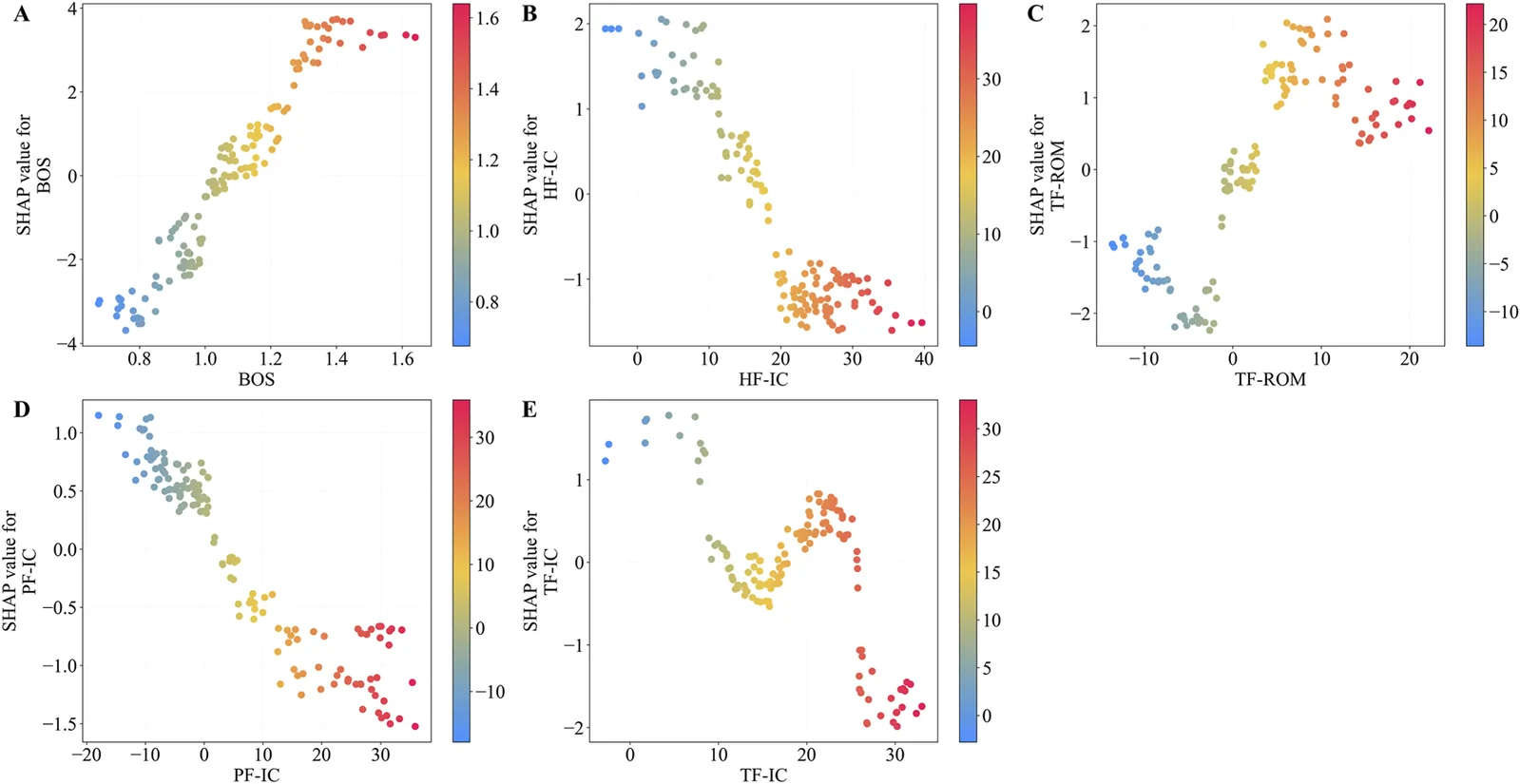
SHAP dependence plots of the XGBoost model. The plots show the relationships between individual feature values and their SHAP attributions to the model output. **(A)** BOS; **(B)** HF-IC; **(C)** TF-ROM; **(D)** PF-IC; **(E)** TF-IC.

To further examine the relationships between the values of these five important variables and their SHAP contributions, dependence plots were generated (Figure 5). Unlike the overall distribution patterns presented by the beeswarm and violin plots, the dependence plots further reveal the continuous relationship between feature values and their SHAP contributions. The results show that for BOS and TF-ROM, lower feature values (blue) correspond to negative SHAP contributions, which favor single-task classification. Higher feature values (red) correspond to positive SHAP contributions, favoring dual-task classification. In contrast, HF-IC, PF-IC, and TF-IC exhibit the opposite pattern. For these features, higher values correspond to negative SHAP contributions, favoring single-task classification.

**FIGURE 6:**
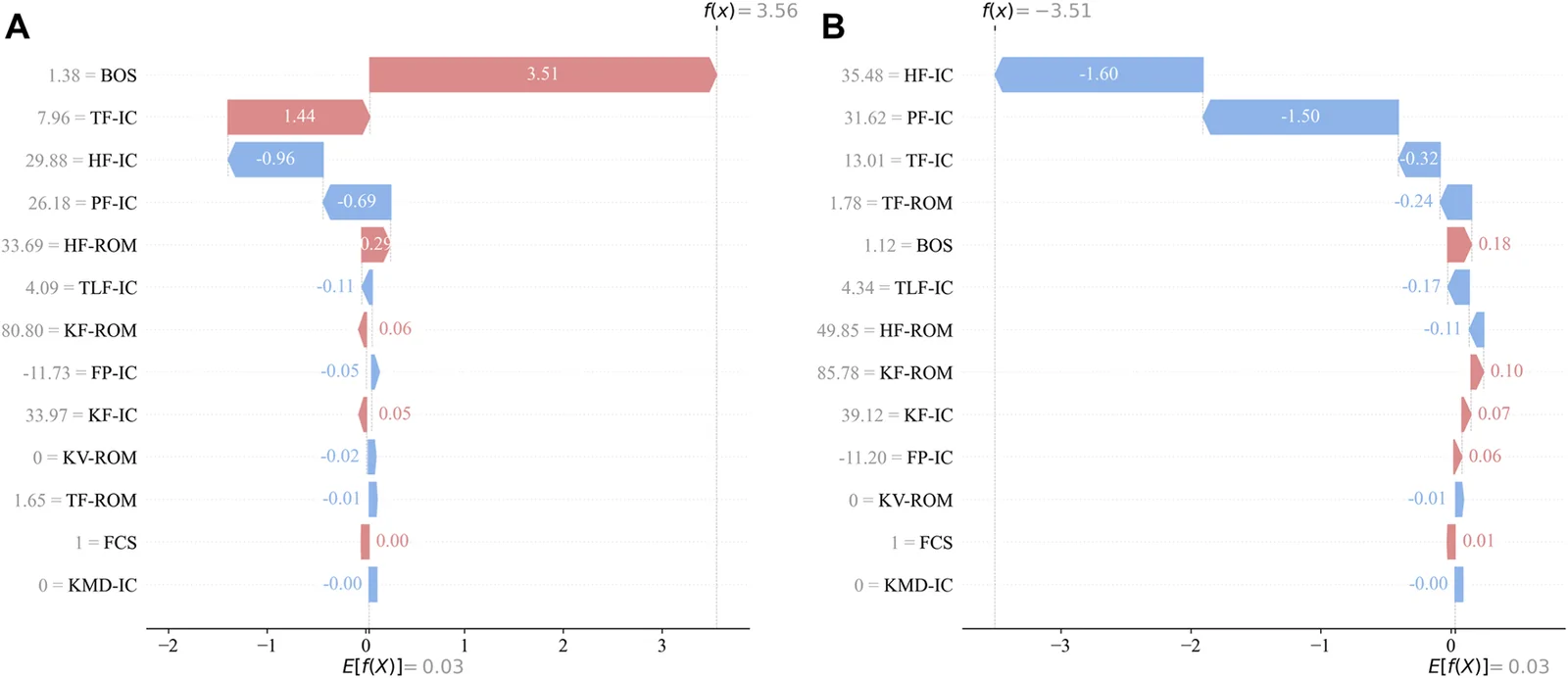
SHAP waterfall plots for selected individual predictions from the held-out test set. Both f(x) and E[f(X)] are raw model outputs (margins); for this binary classifier, they correspond to the log-odds of the dual-task class. **(A)** A dual-task sample correctly classified as dual-task (predicted dual-task probability = 0.972); **(B)** a single-task sample correctly classified as single-task (predicted dual-task probability = 0.029). A probability threshold of 0.50 was used.

To further interpret the model predictions at the individual level, one single-task sample and one dual-task sample were selected from the held-out test set for SHAP waterfall analysis (Figure 6). Within each condition, the correctly classified sample whose predicted dual-task probability was closest to the median predicted probability for that condition was selected. The SHAP waterfall plots visualize the positive and negative contributions of individual features to the model prediction for each sample. Red bars represent features that favor dual-task classification, whereas blue bars represent features that favor single-task classification. The length of each bar indicates the magnitude of the feature contribution. As shown in Figure 6A, the raw model output for the dual-task sample was f(x) = 3.56, corresponding to a predicted dual-task probability of 0.972. BOS (SHAP value = 3.51) and TF-IC (SHAP value = 1.44) were the primary positive contributors, whereas HF-IC (SHAP value = −0.96) and PF-IC (SHAP value = −0.69) contributed negatively. As shown in Figure 6B, the raw model output for the single-task sample was f(x) = −3.51, corresponding to a predicted dual-task probability of 0.029. HF-IC (SHAP value = −1.60) and PF-IC (SHAP value = −1.50) were the primary negative contributors, driving the prediction toward the single-task class, whereas BOS (SHAP value = 0.18) showed only a weak positive contribution. Together, these two examples show how feature contributions combined to form the final prediction for each sample.

## 4 Discussion

When constructing ML predictive models, selecting the optimal model requires balancing accuracy, stability, interpretability, computational efficiency, and data availability (Traidl et al., 2025). Model-data fit and generalization capabilities should also be considered (Riley and Collins, 2023). In this study, four ML models were developed to distinguish between single-task and dual-task jump-landing conditions: XGBoost, Random Forest, SVM, and Logistic Regression. These models were systematically compared using five metrics: AUC, accuracy, recall, precision, and F1-score. XGBoost achieved the highest mean complete outer out-of-fold AUC and the highest mean values for the other four development-set performance metrics. The corrected paired comparisons showed that all three mean outer-fold paired AUC differences were positive. Taken together, these development-set results indicate that XGBoost showed the best overall classification performance among the four algorithms evaluated in this study, with its AUC advantage also supported by the corrected paired comparisons. Considering both model performance and modeling characteristics, Logistic Regression is a generalized linear model. It is widely used as a baseline method due to its good interpretability. However, Logistic Regression assumes a linear relationship between features and labels. This makes it difficult to capture the complex nonlinear relationships between different joints and planes during jump-landing movements. A review by Leckey et al. (2025) found that in sports injury risk prediction, Logistic Regression outperformed other ML methods in only some studies. This suggests that the predictive performance of linear models may be limited under complex, nonlinear feature structures. Although SVM can construct nonlinear decision boundaries through kernel functions, its performance depends on the kernel choice and associated hyperparameters (Cervantes et al., 2020). In the present study, SVM achieved a lower mean development-set AUC than XGBoost under the prespecified RBF kernel and hyperparameter search space. This finding suggests that SVM was less well suited to the feature structure of the present dataset under the current analytical setting. Random Forest relies on a Bagging ensemble strategy. It can effectively reduce variance and handle nonlinear relationships. However, its decision trees are built relatively independently. There is no sequential correction mechanism between trees based on the prediction errors of the previous round (Breiman, 2001). In complex jump-landing movements, multidimensional kinematic variables such as HF-IC and TF-IC may exhibit complex nonlinear relationships. As a result, Random Forest cannot iteratively refine residual errors in the same manner as boosting-based methods. In the present development-set analysis, Random Forest had a lower mean AUC than XGBoost.

In contrast, XGBoost introduces L1 and L2 regularization terms into the objective function. This effectively constrains model complexity. This regularization can reduce the risk of overfitting (Chen and Guestrin, 2016). Its iterative boosting mechanism achieves sequential correction by fitting residuals round by round. Each new tree fits the residuals from the previous prediction round. XGBoost also introduces the second-order gradient of the loss function to guide tree construction. This makes the selection of split directions more precise. This mechanism allows XGBoost to model nonlinear multivariable relationships in jump-landing biomechanics (Chen and Guestrin, 2016). Combined with feature contribution explanation frameworks like SHAP, XGBoost can quantify the specific contribution of each biomechanical variable to an individual prediction. This provides a clear basis for explaining model classification results and the contributions of key biomechanical features. These modeling characteristics provided the rationale for evaluating XGBoost as a candidate algorithm. Its observed performance advantage over the comparator algorithms was supported by the development-set model comparisons and paired AUC analyses described above. This finding is consistent with previous sports-related prediction and biomechanical classification studies. It further supports the hypothesis proposed in this study that XGBoost would achieve better classification performance.

Motor control and cognitive processing involve independent neural pathways, but they share information processing resources in the brain (Leone et al., 2017). When individuals perform motor and cognitive tasks simultaneously, an information processing bottleneck occurs, leading to a performance decline in either task. In this study, the XGBoost-SHAP analysis showed that the five core features contributing most to classification were BOS, HF-IC, TF-ROM, PF-IC, and TF-IC. Compared with the single-task condition, dual-task landing showed decreasing trends in HF-IC, TF-IC, and PF-IC, alongside increasing trends in BOS and TF-ROM. Dai et al. (2018) reported a significant decrease in knee flexion angle at IC during a secondary cognitive task. This change was accompanied by an increased peak vertical GRF and a reduced jump height, which are landing characteristics previously discussed in injury-related biomechanical research. This suggests that cognitive tasks are associated with alterations in landing mechanics that have previously been discussed in ACL-related biomechanical research; however, ACL loading was not directly measured in the present study. Imai et al. (2022) used the same two-digit mental addition paradigm to examine jump-landing and also found decreased hip flexion and increased ankle plantarflexion at IC under dual-task conditions. These results are highly consistent with the directional changes of HF-IC and PF-IC in this study. This is consistent with previous reports that cognitive dual-tasking is associated with reduced hip and knee flexion and with alterations in tibial rotation and landing stance (González-Millán et al., 2024). During cognitive-motor dual-task execution, motor control is not fully automated. It requires the high-level central nervous system to flexibly integrate and coordinate motor and cognitive networks hierarchically. When dual-task demands exceed the processing capacity of the central nervous system, performance may decline in the cognitive task, the motor task, or both. The pattern of decline depends on how attentional resources are allocated (Leone et al., 2017). In this study, PF-IC was identified as an important feature in the SHAP analysis and showed greater plantarflexion under the dual-task condition. This suggests that the cognitive task may draw on attentional resources required for fine control of foot landing posture. Greater ankle plantarflexion at IC may indicate a more anterior foot-contact pattern (Chen et al., 2024; Vendrig et al., 2025). Together with the decreases in HF-IC and TF-IC, this pattern may be related to competition for attentional resources required for postural control. This pattern may reflect altered neural regulation of hip, ankle, and trunk postures, consistent with the competitive allocation of neurocognitive resources. In jump-landing scenarios, cognitive load may reduce the active control of lower limb joints at initial contact by drawing on the shared attentional resources required for postural regulation (Vendrig et al., 2025). This may reflect a more reactive motor-control strategy during dual-tasking. Competition for attentional resources may reduce hip control and may be associated with less active hip and trunk flexion during landing. The observed kinematic changes may alter lower-limb joint energy absorption (Decker et al., 2003). However, joint kinetics were not directly measured in this study. Therefore, whether such changes in energy absorption occurred requires further investigation. Accordingly, increased ACL loading, an ankle-dominant landing strategy, and redistribution of joint energy absorption should be regarded as hypotheses rather than outcomes directly measured in this study. Overall, these five features identified by XGBoost-SHAP constitute the core feature set that describes differences between the two task conditions. The dual-task condition showed a distinct kinematic pattern characterized by altered proximal and distal joint postures. This supports the hypothesis that explainable ML can identify changes in hip and ankle joint postures at initial contact under dual-task conditions.

Additionally, BOS increased significantly under the dual-task condition and was the highest-ranked input feature in the SHAP analysis of the fitted model. This finding suggests that a higher BOS ratio may reflect a compensatory strategy for maintaining postural stability under cognitive interference. In human motor control, BOS is a key factor for maintaining the stability of the center of mass projection (Bonnet, 2012). Under dual-task conditions, the central nervous system may favor postural adjustments that prioritize stability (Mohammadi-Rad et al., 2016). However, a higher BOS ratio does not necessarily improve impact absorption, but may instead reflect a trade-off between postural stability and shock attenuation. Previous literature shows that a narrower BOS reduces dynamic stability (Donelan et al., 2004). Under dual-task conditions, participants showed a higher BOS ratio. This may reflect a trade-off between movement efficiency and postural control (Woollacott and Shumway-Cook, 2002). More importantly, XGBoost-SHAP identified increased BOS and decreased HF-IC as two important features. These changes may reflect a compensatory adaptation. Individuals may widen the BOS to maintain balance when peripheral postural control decreases during dual-task execution. This may be related to competition for attentional resources between the cognitive task and proximal joint control (Woollacott and Shumway-Cook, 2002; Shinya et al., 2011). During the initial landing phase, an increased BOS may represent a spatial compensatory strategy for maintaining stability under dual-task conditions rather than necessarily indicating improved impact attenuation. Therefore, XGBoost-SHAP identified five core features among the 13 input features and quantified their contributions to the model output. This helps explain how the model used these features to distinguish the two task conditions. However, SHAP values quantify feature contributions within the fitted model and do not establish direct biomechanical or physiological causation (Janzing et al., 2020). When predictors are correlated, SHAP attribution may be shared or redistributed among related features; therefore, individual SHAP rankings should not be interpreted as independent biomechanical effects.

This study provides meaningful findings, but it has several limitations. First, the participants were all physically active young men, and the tests were conducted under laboratory conditions using a mental arithmetic task. Therefore, the findings may not generalize to female athletes, older adults, injured populations, elite athletes, or sport-specific visual decision-making contexts without external validation. Second, because no corresponding cognitive single-task condition was included, a formal cognitive dual-task cost could not be calculated; response time and actual task prioritization were not measured, limiting evaluation of the magnitude and consistency of the imposed cognitive demand. Finally, this study did not include prospective injury outcomes, direct measures of ACL loading, an injured comparison group, or an external dataset for validation. Accordingly, these features should be interpreted as descriptors of task-condition differences rather than validated injury-risk indicators, and any injury-related or clinical relevance requires prospective validation.

## 5 Conclusion

XGBoost demonstrated the highest discriminative performance among the four ML algorithms evaluated in this study for classifying single-task and dual-task jump-landing conditions. XGBoost uses its regularization mechanism to constrain model complexity and reduce the risk of overfitting. Additionally, its round-by-round residual fitting mechanism can model nonlinear multi-parameter relationships. Combined with SHAP, the model identified five core features from the biomechanical variables. Under the dual-task condition, these core features included increased BOS, decreased HF-IC, decreased PF-IC, increased TF-ROM, and decreased TF-IC. These changes characterize the biomechanical differences between the two experimental task conditions. Ultimately, the present explainable ML model identified the key biomechanical features that distinguish the two task conditions and quantified their contributions to the model output. The model classifies task condition rather than injury status. Therefore, without prospective validation, these features should not be interpreted as injury-risk markers or screening criteria. In addition, external validation is required before the findings can be generalized to other populations and task contexts.

## Data Availability

The original contributions presented in the study are included in the article/Supplementary Material, further inquiries can be directed to the corresponding author.
